# Subclinical left ventricular deformation and microvascular dysfunction in T2DM patients with and without peripheral neuropathy: assessed by 3.0 T cardiac magnetic resonance imaging

**DOI:** 10.1186/s12933-023-01981-7

**Published:** 2023-09-21

**Authors:** Xue-Ming Li, Rui Shi, Meng-Ting Shen, Wei-Feng Yan, Li Jiang, Chen-Yan Min, Xiao-Jing Liu, Ying-Kun Guo, Zhi-Gang Yang

**Affiliations:** 1 Department of Radiology, West China Hospital, Sichuan University, 37# Guo Xue Xiang, Chengdu, Sichuan China; 2 Laboratory of Cardiovascular Diseases, Regenerative Medicine Research Center, West China Hospital, Sichuan University, 37# Guo Xue Xiang, Chengdu, Sichuan China; 3 Department of Radiology, Key Laboratory of Birth Defects and Related Diseases of Women and Children of Ministry of Education, West China Second University Hospital, Sichuan University, 20# South Renmin Road, Chengdu, Sichuan China

**Keywords:** Type 2 diabetes mellitus, Diabetic peripheral neuropathy, Left ventricular strains, Perfusion, Magnetic resonance imaging

## Abstract

**Background:**

Diabetic peripheral neuropathy (DPN) has been shown to be independently associated with cardiovascular events and mortality. This study aimed to evaluate changes in left ventricular (LV) microvascular perfusion and myocardial deformation in type 2 diabetes mellitus (T2DM) patients with and without DPN, as well as to investigate the association between myocardial perfusion and LV deformation.

**Methods:**

Between October 2015 and July 2022, one hundred and twenty-three T2DM patients without DPN, fifty-four patients with DPN and sixty age‑ and sex‑matched controls who underwent cardiovascular magnetic resonance imaging were retrospectively analyzed. LV myocardial perfusion parameters at rest, including upslope, time to maximum signal intensity (TTM), max signal intensity (max SI), and myocardial strains, including global radial, circumferential and longitudinal strain (GRS, GCS and GLS, respectively), were calculated and compared among the groups with One‑way analysis of variance. Univariable and multivariable linear regression analyses were performed to explore the independent factors influencing LV myocardial perfusion indices and LV strains in diabetes.

**Results:**

The LV GLS, upslope and max SI were significantly deteriorated from controls, through patients without DPN, to patients with DPN (all P < 0.001). Compared with controls, TTM was increased and LV GRS and GCS were decreased in both patient groups (all P < 0.05). Multivariable regression analyses considering covariates showed that DPN was independently associated with reduced upslope, max SI and LV GLS (β = − 0.360, − 2.503 and 1.113, p = 0.021, 0.031 and 0.010, respectively). When the perfusion indices upslope and max SI were included in the multivariable analysis for LV deformation, DPN and upslope (β = 1.057 and − 0.870, p = 0.020 and 0.018, respectively) were significantly associated with LV GLS.

**Conclusion:**

In patients with T2DM, there was more severe LV microvascular and myocardial dysfunction in patients with complicated DPN, and deteriorated subclinical LV systolic dysfunction was associated with impaired myocardial circulation.

## Background

As one of the cardiovascular complications of diabetes, diabetic cardiomyopathy leads to an increased incidence of heart failure and mortality in the general population [1]. Diabetic peripheral neuropathy (DPN) is the most prevalent microvascular complication in individuals with diabetes and is a significant cause of disability and loss of quality of life [2, 3]. It has been shown to be independently associated with cardiovascular events and mortality [4, 5]; however, the mechanisms behind this link are still not clear. Although previous studies have shown that there are abnormalities in cardiac structures and function in diabetic rats and T1DM patients with neuropathy [6, 7], whether there is cooccurrence of myocardial microvascular dysfunction and its association with myocardial function in T2DM patients with DPN is unclear.

Cardiovascular magnetic resonance (CMR) imaging has been increasingly used to comprehensively evaluate myocardial microcirculation as well as cardiac structure and function with high reproducibility. First-pass CMR imaging provides the ability to noninvasively evaluate myocardial perfusion during the transit of gadolinium and has been increasingly used in recent years to detect microvascular dysfunction [8, 9]. The recently developed technology of CMR feature tracking (CMR-FT) can reliably quantify myocardial strain with tissue voxel motion tracking [10]. It has been well established as a sensitive technique for evaluating subclinical left ventricular (LV) systolic dysfunction than LV ejection fraction (LVEF) [10–12] and can predict all-cause mortality and composite cardiovascular endpoints in patients with various heart diseases [13].

Therefore, this study aimed to quantitively evaluate myocardial microcirculation and LV function using CMR first-pass perfusion and CMR-FT technologies in T2DM patients with and without DPN, as well as to investigate the association between myocardial perfusion and LV strains.

## Methods

### Study population

**Fig. 1:**
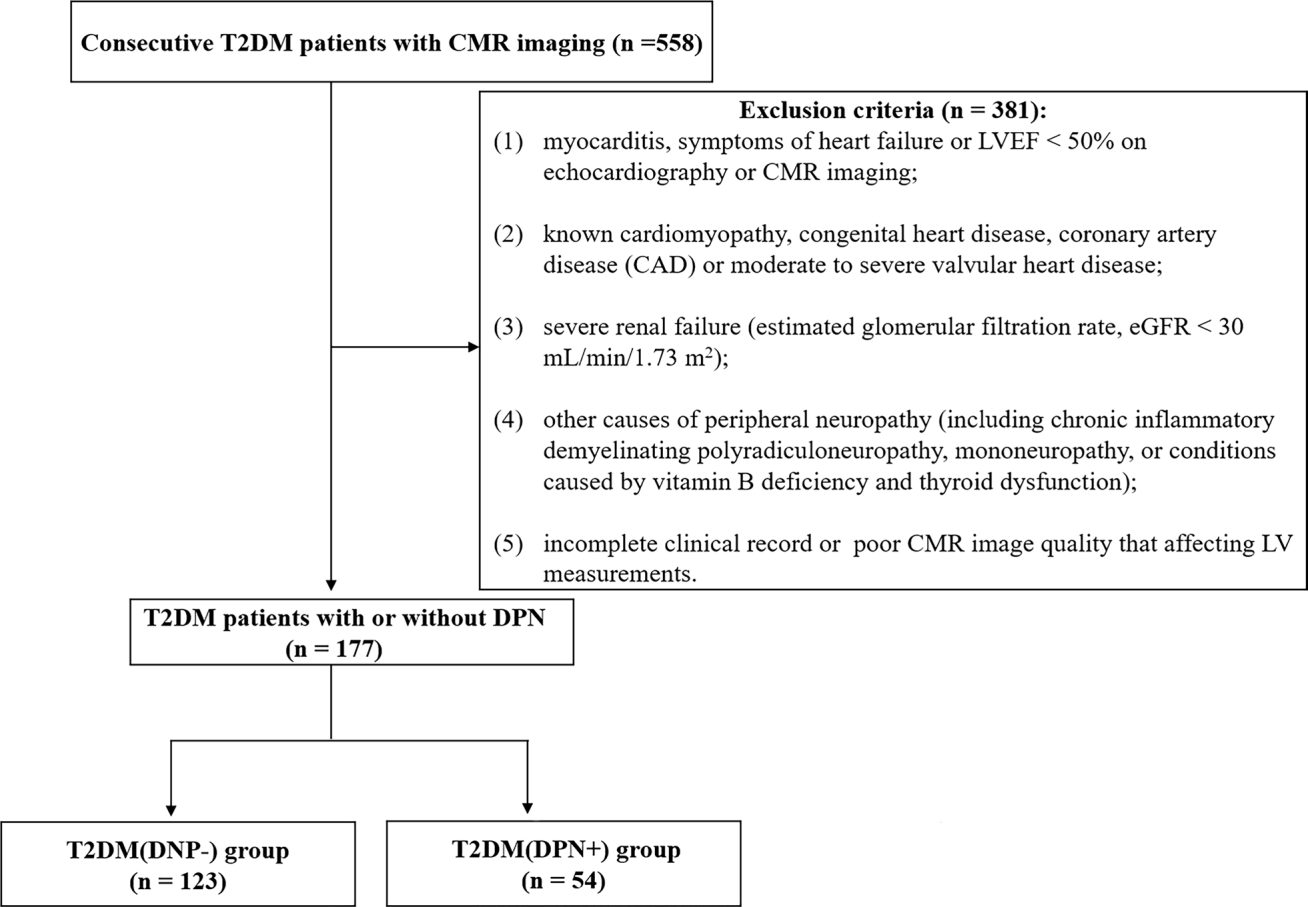
Flowchart of the cohort study. *T2DM* type 2 diabetes mellitus, *CMR* cardiac magnetic resonance, *DPN* diabetes peripheral neuropathy, *LVEF* left ventricular ejection fraction

From October 2015 to July 2022, 558 T2DM patients who had undergone CMR examinations were initially screened. Patients were diagnosed with T2DM according to the diagnostic criteria of the American Diabetes Association guideline (2019) [14], and DPN was diagnosed clinically based on the 2017 diagnostic methods proposed by the American Diabetes Association [15]. The main exclusion criteria were as follows: (1) myocarditis, symptoms of heart failure or LVEF < 50% based on echocardiography or CMR imaging; (2) known cardiomyopathy, congenital heart disease, coronary artery disease (CAD) or moderate to severe valvular heart disease (confirmed by electrocardiogram, echocardiography, angiography, coronary computed tomographic angiography or CMR); (3) severe renal failure (estimated glomerular filtration rate, eGFR < 30 mL/min/1.73 m^2^); (4) other causes of peripheral neuropathy (including chronic inflammatory demyelinating polyradiculoneuropathy, mononeuropathy, or conditions caused by vitamin B deficiency and thyroid dysfunction); and (5) incomplete clinical record or poor CMR image quality that affecting LV measurements. Finally, one hundred seventy-seven T2DM patients, including 123 patients without DPN (69 males and 54 females, mean age 56.5 ± 9.7 years) and 54 patients with DPN (35 males and 19 females, mean age 56.2 ± 9.5), were eligible for this study. A detailed flow chart of the present study is presented in Fig. 1. Additionally, 60 age- and sex-matched individuals (30 males and 30 females; mean age 55.9 ± 9.9 years) were enrolled to serve as the control group. They underwent the same CMR examination for health physical examination, and did not have hypertension, diabetes, impaired glucose tolerance as well as the aforementioned exclusion for T2DM.

This study complied with the Declaration of Helsinki and was approved by the Biomedical Research Ethics Committee of our hospital. Written informed consent was waived due to the retrospective nature of the study.

### CMR protocol

CMR imaging was performed with a 3 T MRI imaging unit MAGNETOM Skrya or TrioTim (Siemens Medical Solutions, Erlangen, Germany). With subjects in the supine position, respiratory and electrocardiography gating were used throughout the scanning process. The short-axis cine images from apex to the base of the heart and long axis 4- and 2-chamber cine images were acquired with a balanced steady-state free precession sequence (repetition time [TR] = 3.4 ms or 2.81 ms, echo time [TE] = 1.22 ms, flip angle = 50° or 40°, slice thickness = 8 mm, field of view [FOV] = 340 × 285 mm^2^ or 250 × 300 mm^2^, matrix size = 256 × 166 or 208 × 139). Twenty-five frames were reconstructed per breath-hold acquisition.

The first-pass myocardial perfusion images were acquired from three short-axis slices (basal, midventricular, and apical level) and a long-axis 4-chamber image with inversion recovery-prepared echo-planar sequence (TR/TE, 163.7/1.12 ms; flip angle, 10°; FOV, 270 mm × 360 mm; matrix size, 106 × 192) after intravenous injection of 0.2 mL/kg gadolinium contrast through the cubital vein at a rate of 2.5–3.0 mL/s, followed by 20 mL of saline. Then, late gadolinium enhancement (LGE) images in the entire LV short-axis stack and from the 2-, 3- and 4-chamber views were acquired to exclude patients with infarct LGE and identify patients with non-infarct LGE after 10–15 min of contrast administration by segmented-turbo-FLASH–phase-sensitive inversion recovery (PSIR) sequence (TR/TE, 300 ms/1.44 ms or 750 ms/1.18 ms, flip angle, 40°, slice thickness, 8 mm, FOV, 275 × 400 mm or 400 × 270 mm, and matrix size = 256 × 184).

### CMR image analysis

#### LV volumetric and functional analysis

The offline commercial software (cvi^42^, v.5.11.2; Circle Cardiovascular Imaging, Inc., Calgary, Canada) was utilized to analyze the CMR images by two radiologists with more than 5 years of experience in CMR who were blinded to the clinical data. First, the LV endo- and epicardial contours were semi-automatically delineated at end-diastole and end-systole on a stack of short-axis cine images, and then LV cardiac geometry and function were automatically calculated, including end-diastolic volume (EDV), end-systolic volume (ESV), stroke volume (SV), LVEF and LV mass (LVM). LVM, LVEDV, LVESV, and LVSV were indexed to body surface area (BSA) and represented as LVMI, LVEDVI, LVESVI and LVSVI, respectively. The papillary muscles and moderator bands were excluded from LVM and included in LV volume. The LV remodeling index was calculated as the ratio of LV mass to LVEDV.

#### LV microvascular perfusion analysis

**Fig. 2:**
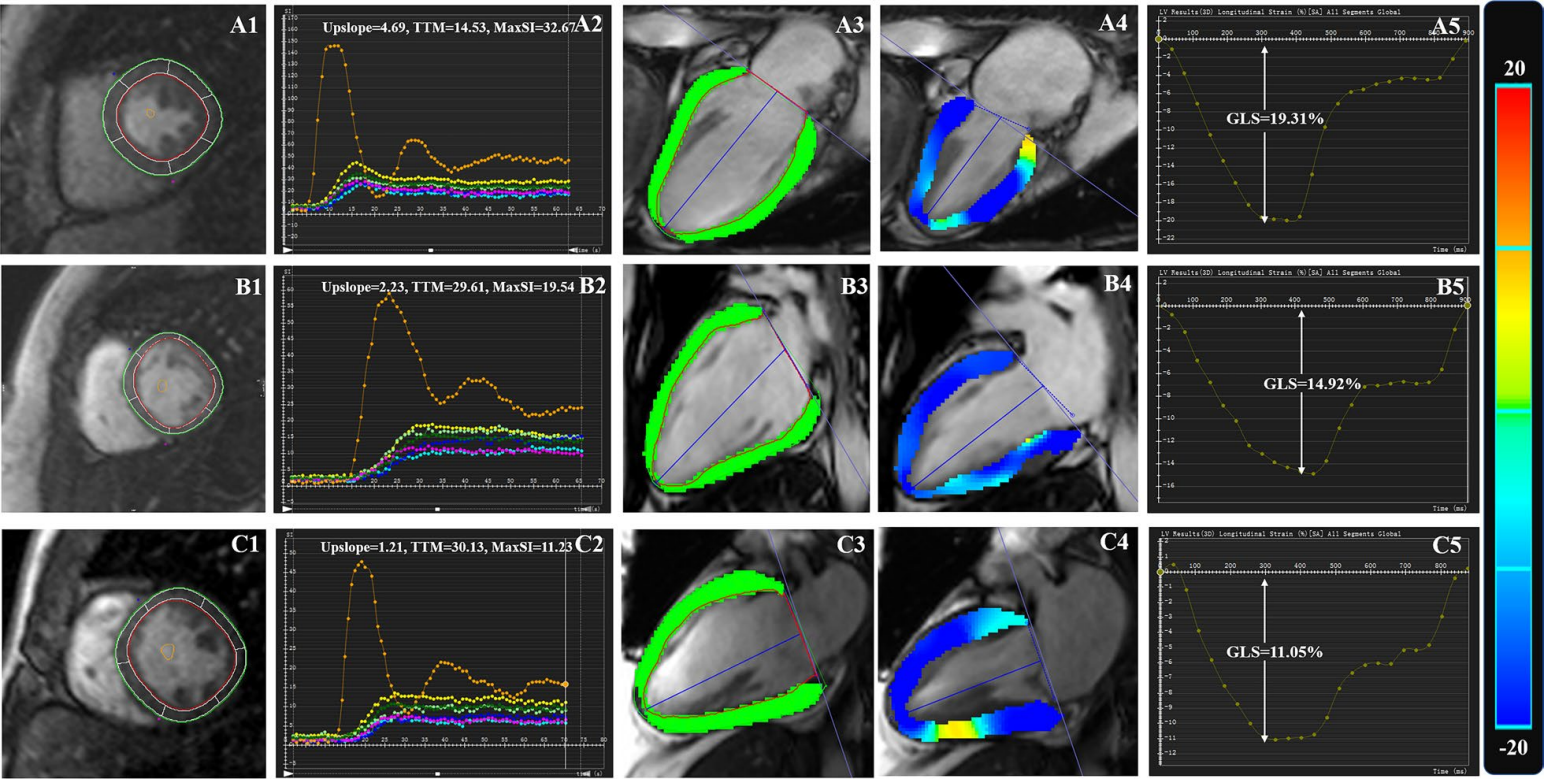
Representative first-pass myocardial perfusion MR images (**A1**, **B1** and **C1**), signal intensity-time curves (**A2**, **B2** and **C2**) obtained from the left mid‑ventricular slice, and longitudinal stain pseudocolour images (**A3–4**, **B3–4**, **C3–4**) and curves (**A5**, **B5**, **C5**) in a normal control (**A1–5**), a T2DM patient without DPN (**B1–5**), and a T2DM patient with DPN (**C1–5**). **A3**, **B3** and **C3** end diastole; **A4**, **B4** and **C4** end systole; **A5**, **B5**, **C5** LV global longitudinal peak strain curve. *T2DM* type 2 diabetes mellitus, *DPN* diabetic peripheral neuropathy, *max SI* maximum signal intensity, *TTM* time-to-maximum signal intensity, *GLS* global longitudinal strain

For microvascular perfusion analysis, the endo- and epicardial contours and a region of interest in the LV blood pool were manually drawn in the first-pass perfusion images of the basal, middle and apical short-axis slices (Fig. 2A1, B1, C1). Subsequently, a myocardial signal intensity-time curve was generated, and the LV semiquantitative perfusion parameters, including upslope, maximum signal intensity (max SI) and time to maximum signal intensity (TTM), were automatically obtained (Fig. 2A2, B2, C2) for each myocardial segment (16 segments, based on the Bull’s eye plot). Subsequently, the global myocardial perfusion indices were calculated for each subject by averaging the regional values of the 16 myocardial segments. The LGE images was categorized by 2 observers in combination into 3 patterns, that is none, infarct, or non-infarct patterns [16].

#### LV strain analysis

For LV myocardial deformation analysis, the endo- and epicardial contours of the left ventricle were semi-automatically outlined at the end diastolic phase in the short-axis and long-axis 2- and 4-chamber cine images in the 3-dimensional (3D) tissue tracking module by the above software. The LV global radial (GRS), circumferential (GCS) and longitudinal (GLS) strain were obtained automatically (Fig. 2A3–5, B3–5, C3–5). The radial strain had a positive value, as it reflects myocardial thickening, while circumferential and longitudinal strain was negative due to myocardium shortening when the chamber wall contracted.

#### Reproducibility of LV strain and first-pass myocardial perfusion parameters

After 1 month, 45 randomly selected cases were remeasured by an experienced original investigator to determine the intraobserver variability in LV global strain and first-pass myocardial perfusion parameters. The same population was evaluated by another investigator who was blinded to the results of the first investigator and clinical data to determine the interobserver variability.

### Statistical analysis

Continuous variables were examined for normality with the Shapiro‒Wilk test. Normally distributed variables are expressed as the mean ± standard deviation (SD) and nonnormal variables as the median (25–75% interquartile range). The differences among the three groups were assessed using one-way analysis of variance (ANOVA) with post hoc Bonferroni correction or Kruskal‒Wallis tests. The duration of T2DM and HbA1c levels were compared between the patient groups by Mann–Whitney U test. Categorical variables are expressed as numbers (percentages) and were compared using Fisher’s exact test or the chi-square test as appropriate. Univariable analysis was conducted to identify the variables related to LV microcirculation and deformation. Variables with a p value < 0.1 in univariable analysis as well as diabetes duration were included in multivariable linear regression analyses to determine the effects of imaging and clinical variables on myocardial circulation and cardiac systolic function in patients with T2DM. Diabetes duration was categorized as long (> 5 years) and short (≤ 5 years) term duration when it was included in the univariable and multivariable analysis. Inter- and intraobserver agreements were determined by the evaluation of intraclass correlation coefficients (ICCs). All statistical analyses were performed with SPSS version 23.0 (IBM, Armonk, New York, USA), and two-tailed *p* values < 0.05 were considered statistically significant.

## Results

### Bassline participant characteristics

The main clinical characteristics of the T2DM patients and control subjects are shown in Table 1. The diabetic duration was significantly longer and HbA1c level was significantly higher in the patients with DPN than in those without DPN (all p < 0.05), and there was a significantly higher incidence of dyslipidemia (61.1% vs. 40.7%, p = 0.0014), retinopathy (33.3% vs. 4.9%, p < 0.001) and chronic kidney disease (CKD) (23.0% vs. 13.0%, p < 0.001) in patients with DPN. No significant differences in triglyceride, total cholesterol, high-density lipoprotein, low-density lipoprotein or eGFR levels were detected among the three groups. In addition, statin and insulin use were significantly higher in the patient group with DPN (p = 0.013 and < 0.001, respectively) than in those without DPN.

**Table 1:** Baseline characteristics of the study cohort

	Controls (n = 60)	T2DM (DPN−) (n = 123)	T2DM (DPN+) (n = 54)
Male, n (%)	30 (50.0)	69 (56.1)	35 (64.8)
Age (years)	55.9 ± 9.9	56.5 ± 9.7	56.2 ± 9.5
BMI (kg/m^2^)	22.81 ± 2.38	24.62 ± 3.48*	24.49 ± 3.31*
BSA (m^2^)	1.72 ± 0.19	1.71 ± 0.16	1.73 ± 0.17
Rest HR (beats/min)	71.6 ± 11.5	73.9 ± 11.2	75.1 ± 9.9
Systolic blood pressure (mmHg)	118 (106, 123)	127 (119, 140) *	128 (120, 144)*
Diastolic blood pressure (mmHg)	73.4 ± 8.9	80.4 ± 10.2*	79 (73, 85)*
Diabetic duration (years)	NA	5 (2, 10)	8 (3, 14.7)^&^
Dyslipidemia, n (%)	NA	50 (40.7)	33 (61.1)^&^
Retinopathy, n (%)	NA	6 (4.9)	18 (33.3)^&^
CKD, n (%)	NA	17( 13.8)	12 (22.2)^&^
Laboratory data			
FBG (mmol/L)	5.11 (4.79, 5.72)	7.32 (6.10, 8.88)*	7.60 (6.20, 11.4)*
HbA1c (%)	NA	6.6 (6.2, 7.7)	7.25 (6.8, 9.25)^&^
Plasma triglycerides (mmol/L)	1.20 (0.99, 1.79)	1.27 (0.91, 1.97)	1.24 (1.00, 2.00)
Total cholesterol (mmol/L)	4.55 ± 0.79	4.32 ± 0.92	4.25 (3.66, 5.27)
HDL (mmol/L)	1.29 (1.09, 1.63)	1.17 (1.00, 1.49)	1.23 (0.92, 1.44)
LDL (mmol/L)	2.48 (1.93, 3.03)	2.56 (2.18, 3.14)	2.43 (1.88, 3.48)
eGFR (mL/min/1.73 m^2^)	91.44 ± 18.74	91.40 ± 18.32	93.29 ± 21.67
Medications, n (%)			
Statin	NA	29 (23.6)	21 (38.9)^&^
Biguanides	NA	72 (58.5)	34 (63.0)
Sulfonylureas	NA	30 (24.4)	15 (27.8)
α-Glucosidase inhibitor	NA	48 (39.0)	26 (48.1)
GLP-1/DDP-4 inhibitor	NA	11 (8.9)	7 (13.0)
Insulin	NA	27 (22.0)	32 (59.3)^&^
ACEI/ARB	NA	33 (26.8)	11 (20.4)
β-Blocker	NA	12 (9.8)	5 (9.3)
Calcium channel blocker	NA	35 (28.5)	15 (27.8)

The values are the mean ± SD, numbers in the brackets are percentages

*T2DM* type 2 diabetes mellitus, *DPN* diabetic peripheral neuropathy, *BMI* body mass index, *BSA* body surface area, *HR* heart rate, *CKD* chronic kidney disease, *FBG* fasting blood glucose, *HbA1c* glycated hemoglobin, *HDL* high-density lipoprotein cholesterol, *LDL* low-density lipoprotein cholesterol, *eGFR* estimated glomerular filtration rate, *ACEI* angiotensin converting enzyme inhibitor, *ARB* angiotensin II receptor blocker, *GLP-1* glucagon-like peptide-1, *DPP-4* dipeptidyl peptidase-4

*p < 0.05 versus controls

^&^P < 0.05 versus T2DM (DPN−) group

### Comparison of CMR findings among groups

As demonstrated in Table 2, the LVM (p < 0.001 and = 0.005, respectively), LVMI (p < 0.001 and = 0.005, respectively) and LV remodeling index (p < 0.001 and = 0.005, respectively) were significantly higher in both patient groups than in the controls (all P < 0.05), while they were not significantly different the patient groups (all p > 0.05). The incidence of non-infarct LGE was significantly higher in patients with DPN than those without DPN (37.0% vs. 17.9%, p = 0.008). All the other LV geometric parameters were not significantly different among the three groups (all p > 0.05).

**Table 2:** Comparison of CMR findings among T2DM patients with/without DPN and normal controls

	Controls	T2DM (DPN−)	T2DM (DPN+)
LV geometry			
LVEDV (mL)	123.80 ± 23.27	127.27 ± 26.70	131.81 ± 31.58
LVEDVI (mL/m^2^)	72.20 ± 11.53	74.54 ± 14.55	75.77 ± 16.20
LVESV (mL)	42.96(33.16, 51.85)	45.86 (37.39, 55.16)	43.73 (36.02, 54.63)
LVESVI (mL/m^2^)	25.17 (21.27, 29.29)	26.40 (21.67, 31.22)	24.60 (21.68, 30.69)
LVSV (mL)	79.98 ± 16.19	80.85 ± 17.75	84.13 ± 20.24
LVSVI (mL/m^2^)	46.71 ± 8.56	47.29 ± 9.49	48.43 ± 10.38
Cardiac index (L/min/m^2^)	3.34 ± 0.86	3.49 ± 0.82	3.59 ± 0.74
LVEF (%)	64.66 ± 6.38	63.70 ± 7.02	64.12 ± 7.59
LVM (g)	68.13 (59.61, 82.62)	79.42 (66.53, 95.12)*	90.96 (68.37, 109.01)*
LVMI (g/m^2^)	41.25 (37.73, 46.46)	46.81 (38.41, 53.89)*	49.21 (40.73, 59.78)*
LV remodeling index (g/mL)	0.58 (0.51, 0.64)	0.63 (0.55, 0.74)*	0.66 (0.55, 0.80) *
Non-infarct LGE, n (%)	NA	22 (17.9)	20 (37.0)^&^
Myocardial strain			
GRS (%)	36.94 ± 7.21	33.42 ± 7.04*	32.02 ± 9.77*
GCS (%)	− 21.13 ± 2.53	− 19.99 ± 2.34*	− 19.39 ± 3.25*
GLS (%)	− 15.07 ± 2.56	− 13.24 ± 2.46*	− 11.92 ± 3.29*^&^
Myocardial perfusion			
Upslope	2.69 ± 1.19	2.25 ± 1.18*	1.76 ± 0.60*^&^
TTM (s)	25.73 ± 9.71	31.57 ± 13.39*	31.68 ± 12.86*
Max SI	22.83 ± 8.41	19.97 ± 7.81*	16.04 ± 4.88*^&^

The values are mean ± SD, numbers in the brackets are percentages

*T2DM* type 2 diabetes mellitus, *DPN* diabetic peripheral neuropathy, *LV* left ventricular, *EDV* end diastolic volume, *ESV* end systolic volume, *SV* stroke volume, *EF* ejection fraction, *M* mass, *I* indexed to BSA, *LGE* late gadolinium enhancement, *GRS* global radial strain, *GCS* global circumferential strain, *GLS* global longitudinal strain, *TTM* time to maximum signal intensity, *Max SI* max signal intensity

*p < 0.05 versus controls

^&^P < 0.05 versus T2DM (DPN−) group

The LV upslope and max SI significantly and gradually deteriorated from controls to patients without DPN to patients with DPN (upslope: 2.69 ± 1.19 vs. 2.25 ± 1.18 vs. 17.6 ± 0.60, p < 0.001; Max SI: 22.83 ± 8.41 vs. 19.97 ± 7.81 vs. 16.04 ± 4.88, p < 0.001). In addition, the TTM was higher in both patient groups than in the controls (all P < 0.05), but it was not different between the patient groups (p > 0.05).

The LV GLS declined significantly from controls, to patients without DPN, to patients with DPN (p < 0.001). Compared with the control group, LV GRS and GCS were decreased in both patient groups (both p < 0.05) but were not different between the groups (all p > 0.05).

### Association between first-pass perfusion parameters and risk factors in T2DM patients

The results of univariable and multivariable linear regression analyses for perfusion parameters in T2DM patients are shown in Table 3. In the multivariable analyses after considering covariates, both DPN (β = − 0.360, p = 0.021 and β = − 2.503 and p = 0.031, respectively) and CKD (β = − 0.399, p = 0.043 and β = − 4.057 and p = 0.005, respectively) were significantly associated with reduced upslope and max SI. In addition, dyslipidemia was significantly associated with max SI (β = − 2.569, p = 0.018), and it was not associated with upslope (β = − 0.114, p = 0.104).

**Table 3:** Univariable and multivariable analysis between first-pass perfusion parameters and clinical indices in T2DM patients

	Upslope			Max SI			TTM (s)		
	Univariable	Multivariable		Univariable	Multivariable		Univariable	Multivariable	
	r	β	p	r	β	p	r	β	p
DPN	− 0.487*	− 0.360	0.021	− 3.934*	− 2.503	0.031	− 0.115		
Retinopathy	− 0.289			− 1.374			2.617		
CKD	− 0.402*	− 0.399	0.043	− 4.741*	− 4.057	0.005	− 4.388		
Dyslipidemia	− 0.296*	− 0.114	0.104	− 3.092*	− 2.569	0.018	1.317		
Age (years)	0.002			0.031			− 0.057		
Sex (male = 1)	− 0.840*	− 0.885	< 0.001	− 4.159*	− 3.556	0.001	8.352*	11.073	< 0.001
BMI (kg/m^2^)	− 0.051*	− 0.057	0.007	− 0.252			− 0.045		
HR (beats/min)	0.028*	0.025	< 0.001	0.065			− 0.397*	− 0.346	0.001
SBP (mmHg)	0.002			0.026			− 0.130*	− 0.138	0.022
Smoking	− 0.427*	0.058	0.449	− 1.106			5.263*	− 0.093	0.291
LVM (g)	− 0.013*	0.015	0.861	− 0.069*	0.005	0.951	0.087*	0.020	0.833
HbA1c (%)	− 0.007			0.272			1.121*	0.093	0.224
Diabetic duration (long = 1)	− 0.202	− 0.053	0.438	0.412	− 0.094	0.197	− 2.096	0.076	0.319
Statin	− 0.027			− 1.894			− 0.144		
Biguanides	0.178*	− 0.093	0.171	− 2.590*	− 0.104	0.151	2.689		
Sulfonylureas	− 0.310*	− 0.074	0.285	− 1.860			2.223		
α-Glucosidase inhibitor	− 0.449*	− 0.492	0.001	− 2.908*	− 2.388	0.025	2.569		
GLP-1/DDP-4 inhibitor	0.157			0.406			1.211		
Insulin	− 0.265			− 1.794			0.788		
ACEI/ARB	− 0.001			0.886			− 2.900		
β-Blocker	0.227			0.770			− 6.094*	− 0.042	0.579
Calcium channel blocker	0.120			0.451			− 3.590		
R^2^		0.364			0.235			0.273	

Variables with p < 0.1 in the univariable analysis as well as diabetic duration were included in the multivariable liner regression model

Abbreviations as in Tables 1 and 2; long = 1 means patients with long term duration > 5 years

^*^ p < 0.1

### Association of DPN and first-pass perfusion with LV GLS in T2DM patients

The results of univariable and multivariable linear regression analyses for LV GLS in T2DM patients are shown in Table 4. In the univariable regression analysis, DPN, retinopathy, CKD, sex, smoking, LVM, β-blocker and all the perfusion parameters were significantly correlated with LV GLS (all p < 0.05). In the multivariable analysis including covariates of risk factors, LVM, β-blocker and diabetic duration, DPN (β = 1.113, p = 0.010), CKD (β = 1.223, p = 0.032), sex (β = 1.646, p = 0.001) and were significantly associated with LV GLS. When the perfusion parameters upslope and max SI were added to the multivariable analysis, it showed that DPN (β = 1.057, p = 0.020), CKD (β = 1.539, p = 0.009), sex (β = 1.798, p < 0.001) and upslope (β = − 0.870, p = 0.018) were significantly associated with LV GLS.

**Table 4:** Association of DPN and first-pass perfusion with LV GLS in patients with type 2 diabetes mellitus

	Univariable		Multivariable		Multivariable	
	r	p	β^a^	p	β^b^	p
DPN	1.32	0.004	1.113	0.010	1.057	0.020
Retinopathy	1.496	0.015	0.088	0.235	0.077	0.299
CKD	1.818	0.001	1.223	0.032	1.539	0.009
Dyslipidemia	− 0.18	0.671				
Age (years)	− 0.01	0.651				
Sex (male = 1)	1.99	< 0.001	1.646	0.001	1.798	< 0.001
BMI (kg/m^2^)	0.065	0.300				
HR (beats/min)	− 0.003	0.873				
SBP (mmHg)	0.016	0.228				
Smoking	1.313	0.006	0.032	0.687	0.011	0.890
LVM (g)	0.044	< 0.001	0.018	0.068	0.136	0.118
HbA1c (%)	0.12	0.321				
Diabetic duration (long = 1)	0.05	0.187	0.091	0.196	0.113	0.116
Statin	− 0.027	0.954				
Biguanides	0.178	0.680				
Sulfonylureas	0.276	0.571				
α-Glucosidase inhibitor	− 0.069	0.874				
GLP-1/DDP-4 inhibitor	− 0.066	0.925				
Insulin	0.340	0.450				
ACEI/ARB	0.741	0.130				
β-Blocker	1.552	0.030	1.324	0.045	1.527	0.025
Calcium channel blocker	− 0.172	0.716				
Upslope	− 0.808	< 0.001			− 0.870	0.018
Max SI	− 0.078	0.009			0.095	0.066
R^2^			0.264		0.270	

Abbreviations as in Tables 1 and 2; long = 1 means patients with long term duration > 5 years

^a^Variables with P < 0.1 in the univariable analysis as well as diabetic duration were included in the multivariable analysis

^b^The perfusion parameters upslope and max SI were added as covariates in the multivariable analysis

### Intra-observer and inter-observer variability

Table 5 demonstrates the intra- and interobserver variability for LV deformation and first-pass myocardial perfusion analysis. As demonstrated, the ICCs for intra- and interobserver variabilities were 0.913–0.947 and 0.901–0.913, respectively, for LV deformation and 0.935–0.947 and 0.901–0.937, respectively, for first-pass myocardial perfusion, suggesting excellent agreements for both techniques.

**Table 5:** Intra- and inter- observer variability of perfusion parameters and myocardial strains

	Intra-observer		Inter-observer	
	ICC	95% CI	ICC	95% CI
Upslope	0.947	0.913–0.986	0.937	0.903–0.987
TTM (s)	0.936	0.902–0.978	0.901	0.895–0.943
Max SI	0.935	0.825–0.957	0.912	0.903–0.946
LV GRS	0.913	0.911–0.963	0.902	0.834–0.912
LV GCS	0.934	0.905–0.952	0.901	0.869–0.925
LV GLS	0.947	0.913–0.957	0.913	0.898–0.952

*ICC* intraclass correlation coefficient, *CI* confidence interval; other abbreviations as in Table 2

## Discussion

In this study, we found some important results as follows. LV myocardial perfusion and myocardial deformation were worse in T2DM patients without DPN than in the controls, and they were further deteriorated in patients with DPN. Impaired myocardial microcirculation and DPN were independently associated with subclinical LV systolic dysfunction in patients with T2DM.

The pathophysiology of cardiomyopathy related to T2DM is complex and multifactorial. Among these, coronary microvascular abnormalities, are considered a comprehensive result of endothelial dysfunction, microvascular rarefaction, and perivascular collagen and AGE deposition [1]. A previous study quantifying myocardial perfusion with PET by Murthy et al. demonstrated that microvascular dysfunction carries significant independent prognostic significance for both patients with and without diabetes [17]. The perfusion parameters calculated with CMR perfusion imaging are validated against microsphere measurements and can objectively reflect the degree of microvascular dysfunction [18]. In this study, significantly reduced first-pass resting myocardial perfusion was observed in both T2DM patients without and with DPN compared with controls. In addition, some previous studies have also reported decreased resting perfusion [19, 20], while others showed increased resting myocardial perfusion [21–23]. This discrepancy may be due to different study populations, investigation modalities, and relatively modest sample sizes.

DPN can be considered a part of the continuum of disease-related microvascular complications, which include peripheral neuropathy, nephropathy, retinopathy and diabetic cardiomyopathy, and indicate a systemic rather than organ-specific disease [3, 7, 24]. All microvascular complications of diabetes arise early, and they are related to each other via complex pathological mechanisms [25, 26]. Univariable and multivariable regression analyses revealed that both DPN and chronic kidney disease (CKD) were associated with perfusion impairment, this suggests that the characteristics of the CMR findings observed in this study are not specific to patients with DPN, but may be present in a group of patients with diabetic microvascular complications. It has been well established that microvascular dysfunction plays an important role in the development of DPN [27], and there is substantial evidence of severer microvascular impairment in T2DM with peripheral neuropathy than those without [28]. Our results demonstrated that T2DM patients with DPN had significantly decreased myocardial perfusion compared with both controls and patients without DPN even in the resting state, and more non-infarct LGE in those with DPN, indicating severer myocardial microcirculatory dysfunction in patients with DPN. The study by Baltzis reported that patients with DPN had a higher risk of myocardial ischemia than those without DPN with single-photon emission computed tomographic (SPECT) imaging [29]. In addition, Jende et al. conducted the first study to find a strong association of hsTNT with measures of diffusion-weighted neuroimaging that codifies structural nerve integrity as well as with clinical neuropathy scores and electrophysiological data in T2DM patients with neuropathy [30], which supports the hypothesis that decreased neural blood supply contributes to the deterioration of axons and Schwann cells in diabetic neuropathy [2, 31]. All these results may strongly indicate that cardiac microangiopathy exists in parallel with peripheral microangiopathy, which is an important contributor to DPN in T2DM.

Epidemiologic studies have provided substantial evidence that there is worsened cardiac function and higher cardiovascular mortality in patients with DPN. In a diabetic rat model, there was co-occurrence of myocardial dysfunction and peripheral insensate neuropathy [6]. We found that the subclinical LV systolic dysfunction represented by LV GLS was further reduced in T2DM patients complicated with DPN despite comparable LVEF. These results suggested that there was more severe subclinical LV systolic dysfunction in diabetic patients with DPN. We postulated that the mechanisms for the deterioration of cardiac function in patients with DPN may be attributed to more severe pathological abnormalities in the myocardium, such as various neurohormonal and metabolic abnormalities (e.g., apoptosis, inflammation, oxidative stress and fibrosis), and abnormalities in microvasculature and cardiac remodeling [1–3]. Furthermore, we found that LV GLS, not GRS or GCS, was further reduced in patients with comorbid DPN. This may be because the myocardium producing longitudinal strain mainly exists in the subendocardium and is more sensitive to these pathological changes, causing early and more severe myocardial dysfunction in the subendocardium [10, 13].

Coronary microvascular dysfunction, as a direct cause of myocardial tissue hypoxia, is an important factor involved in the development of diabetic cardiomyopathy. Multivariable regression analyses showed negative association between DPN and LV myocardial perfusion and myocardial function. The negative association between DPN and LV GLS suggested that DPN was an important indicator of LV systolic dysfunction. We found that worsened microvascular dysfunction associated with DPN was negatively associated with LV systolic function in patients with T2DM, which was in line with previous results [9, 32]. In addition, some other studies have found that lower myocardial perfusion reserve on CMR imaging was associated with LV dysfunction [33] and impaired exercise capacity [34]. All these results indicate that there may be a possible mechanistic link between myocardial perfusion impairment and progressive changes in cardiac strain and function in T2DM patients, which may be explained as the disturbance of myocardial microcirculation compromising oxygen delivery and energy utilization, reducing myocardial contractility, and finally limiting LV systolic function [1, 32]. Further trials into medications to increase myocardial microvasculature to prevent heart failure are required.

## Limitations

There are several limitations in our study. First, the sample size in our retrospective study was relatively small, and the results should be verified by studies with larger cohorts or involving multiple centers. Second, invasive coronary angiography and coronary computed tomography angiography were not performed in all patients. However, significant CAD was deemed to be unlikely by comprehensive evaluation of the patients’ clinical history, laboratory results, electrocardiogram and echocardiography, which was supported by the CMR examinations. Third, Considering the contraindications and potential risks associated with cardiac stress test, our study solely evaluated microcirculation function at rest. However, even in the resting state, our results showed impaired myocardial microcirculation in both T2DM patients without and with DPN, and the clinical significance of resting myocardial perfusion assessment warrants further attention and validation. Finally, we could not determine the causal relationship between LV myocardial dysfunction and microvascular dysfunction due to the cross-sectional nature of the study. Further longitudinal studies are needed to explore the potential prognostic value of impaired myocardial perfusion and deformation in T2DM patients with disease complicated by DPN.

## Conclusions

In T2DM patients, there was more severe myocardial microvascular impairment and deformation dysfunction in patients with complicated DPN, and deteriorated subclinical LV systolic dysfunction was associated with myocardial microvascular dysfunction.

## Data Availability

The datasets used in the current study are available from the corresponding author on reasonable request.

## Abbreviations

DPN: Diabetic peripheral neuropathy
T2DM: Type two diabetes mellitus
LV: Left ventricular
CMR: Cardiovascular magnetic resonance
CMR-FT: CMR feature tracking
EDV: End-diastolic volume
ESV: End-systolic volume
LVEF: Left ventricular ejection fraction
LVMI: LV mass index
GRS: Global radial strain
GCS: Global circumferential strain
GLPS: Global longitudinal strain
TTM: Time to maximum signal intensity
max SI: Max signal intensity

## References

[1] Marwick TH, Ritchie R, Shaw JE, Kaye D (2018). Implications of underlying mechanisms for the recognition and management of diabetic cardiomyopathy. J Am Coll Cardiol.

[2] Feldman EL, Callaghan BC, Pop-Busui R, Zochodne DW, Wright DE, Bennett DL (2019). Diabetic neuropathy. Nat Rev Dis Prim.

[3] Iqbal Z, Azmi S, Yadav R, Ferdousi M, Kumar M, Cuthbertson DJ (2018). Diabetic peripheral neuropathy: epidemiology, diagnosis, and pharmacotherapy. Clin Ther.

[4] Brownrigg JR, Davey J, Holt PJ, Davis WA, Thompson MM, Ray KK (2012). The association of ulceration of the foot with cardiovascular and all-cause mortality in patients with diabetes: a meta-analysis. Diabetologia.

[5] Margariti A (2014). Peripheral neuropathy may be a potential risk of cardiovascular disease in diabetes mellitus. Heart.

[6] Marangoni MN, Brady ST, Chowdhury SA, Piano MR (2014). The co-occurrence of myocardial dysfunction and peripheral insensate neuropathy in a streptozotocin-induced rat model of diabetes. Cardiovasc Diabetol.

[7] Hansen GM, Jorgensen PG, Andersen HU, Rossing P, Jensen MT (2021). Relationship between peripheral neuropathy, diastolic function and adverse cardiovascular outcome in individuals with type 1 diabetes mellitus without known cardiovascular disease: results from the thousand & 1 study. Diabetes Obes Metab.

[8] Wang J, Yang ZG, Guo YK, Jiang Y, Yan WF, Qian WL (2023). Incremental effect of coronary obstruction on myocardial microvascular dysfunction in type 2 diabetes mellitus patients evaluated by first-pass perfusion CMR study. Cardiovasc Diabetol.

[9] Li XM, Jiang L, Guo YK, Ren Y, Han PL, Peng LQ (2020). The additive effects of type 2 diabetes mellitus on left ventricular deformation and myocardial perfusion in essential hypertension: a 3.0 T cardiac magnetic resonance study. Cardiovasc Diabetol.

[10] Claus P, Omar AMS, Pedrizzetti G, Sengupta PP, Nagel E (2015). Tissue tracking technology for assessing cardiac mechanics: principles, normal values, and clinical applications. JACC Cardiovasc Imaging.

[11] Wang J, Li Y, Guo YK, Huang S, Shi R, Yan WF (2022). The adverse impact of coronary artery disease on left ventricle systolic and diastolic function in patients with type 2 diabetes mellitus: a 3.0T CMR study. Cardiovasc Diabetol.

[12] Li XM, Yan WF, Jiang L, Shi K, Ren Y, Han PL (2022). Impact of T2DM on right ventricular systolic dysfunction and interventricular interactions in patients with essential hypertension: evaluation using CMR tissue tracking. Cardiovasc Diabetol.

[13] Kalam K, Otahal P, Marwick TH (2014). Prognostic implications of global LV dysfunction: a systematic review and meta-analysis of global longitudinal strain and ejection fraction. Heart.

[14] Chamberlain JJ, Rhinehart AS, Shaefer CF, Neuman A (2016). Diagnosis and management of diabetes: synopsis of the 2016 American diabetes association standards of medical care in diabetes. Ann Intern Med.

[15] Pop-Busui R, Boulton AJ, Feldman EL, Bril V, Freeman R, Malik RA (2017). Diabetic neuropathy: a position statement by the American diabetes association. Diabetes Care.

[16] Schulz-Menger J, Bluemke DA, Bremerich J, Flamm SD, Fogel MA, Friedrich MG (2020). Standardized image interpretation and post-processing in cardiovascular magnetic resonance—2020 update : society for cardiovascular magnetic resonance (SCMR): board of trustees task force on standardized post-processing. J Cardiovasc Magn Reson.

[17] Murthy VL, Naya M, Foster CR, Gaber M, Hainer J, Klein J (2012). Association without diabetes mellitus. Circulation.

[18] Jerosch-Herold M (2010). Quantification of myocardial perfusion by cardiovascular magnetic resonance. J Cardiovasc Magn Reson.

[19] Liu X, Yang ZG, Gao Y, Xie LJ, Jiang L, Hu BY (2018). Left ventricular subclinical myocardial dysfunction in uncomplicated type 2 diabetes mellitus is associated with impaired myocardial perfusion: a contrast-enhanced cardiovascular magnetic resonance study. Cardiovasc Diabetol.

[20] Cai X, Zhang S, Deng D, Li H, Guan X, Fang J (2018). Myocardial perfusion at rest in uncomplicated type 2 diabetes patients without coronary artery disease evaluated by 320-multidetector computed tomography: a pilot study. Medicine.

[21] Picchi A, Limbruno U, Focardi M, Cortese B, Micheli A, Boschi L (2011). Increased basal coronary blood flow as a cause of reduced coronary flow reserve in diabetic patients. Am J Physiol Heart Circ Physiol.

[22] Meyer C, Schwaiger M (1997). Myocardial blood flow and glucose metabolism in diabetes mellitus. Am J Cardiol.

[23] Sorensen MH, Bojer AS, Broadbent DA, Plein S, Madsen PL, Gaede P (2020). Cardiac perfusion, structure, and function in type 2 diabetes mellitus with and without diabetic complications. Eur Heart J Cardiovasc Imaging.

[24] Sacchetta L, Chiriaco M, Nesti L, Leonetti S, Forotti G, Natali A (2022). Synergistic effect of chronic kidney disease, neuropathy, and retinopathy on all-cause mortality in type 1 and type 2 diabetes: a 21-year longitudinal study. Cardiovasc Diabetol.

[25] Dyck PJ, Kratz KM, Karnes JL, Litchy WJ, Klein R, Pach JM (1993). The prevalence by staged severity of various types of diabetic neuropathy, retinopathy, and nephropathy in a population-based cohort: the Rochester diabetic neuropathy study. Neurology.

[26] Ali A, Iqbal F, Taj A, Iqbal Z, Amin MJ, Iqbal QZ (2013). Prevalence of microvascular complications in newly diagnosed patients with type 2 diabetes. Pak J Med Sci.

[27] Roustit M, Loader J, Deusenbery C, Baltzis D, Veves A (2016). Endothelial dysfunction as a link between cardiovascular risk factors and peripheral neuropathy in diabetes. J Clin Endocrinol Metab.

[28] Loader J, Khouri C, Taylor F, Stewart S, Lorenzen C, Cracowski JL (2019). The continuums of impairment in vascular reactivity across the spectrum of cardiometabolic health: a systematic review and network meta-analysis. Obes Rev.

[29] Baltzis D, Roustit M, Grammatikopoulou MG, Katsaboukas D, Athanasiou V, Iakovou I (2016). Diabetic peripheral neuropathy as a predictor of asymptomatic myocardial ischemia in type 2 diabetes mellitus: a cross-sectional study. Adv Ther.

[30] Jende JME, Groener JB, Kender Z, Hahn A, Morgenstern J, Heiland S (2020). Troponin T parallels structural nerve damage in type 2 diabetes: a cross-sectional study using magnetic resonance neurography. Diabetes.

[31] Jende JME, Groener JB, Kender Z, Rother C, Hahn A, Hilgenfeld T (2020). Structural nerve remodeling at 3-T MR neurography differs between painful and painless diabetic polyneuropathy in type 1 or 2 diabetes. Radiology.

[32] Jiang L, Wang J, Liu X, Li ZL, Xia CC, Xie LJ (2020). The combined effects of cardiac geometry, microcirculation, and tissue characteristics on cardiac systolic and diastolic function in subclinical diabetes mellitus-related cardiomyopathy. Int J Cardiol.

[33] Larghat AM, Swoboda PP, Biglands JD, Kearney MT, Greenwood JP, Plein S (2014). The microvascular effects of insulin resistance and diabetes on cardiac structure, function, and perfusion: a cardiovascular magnetic resonance study. Eur Heart J Cardiovasc Imaging.

[34] Gulsin GS, Henson J, Brady EM, Sargeant JA, Wilmot EG, Athithan L (2020). Cardiovascular determinants of aerobic exercise capacity in adults with type 2 diabetes. Diabetes Care.

